# Editorial: ViTac: Integrating Vision and Touch for Multimodal and Cross-Modal Perception

**DOI:** 10.3389/frobt.2021.697601

**Published:** 2021-05-07

**Authors:** Shan Luo, Nathan F. Lepora, Uriel Martinez-Hernandez, Joao Bimbo, Huaping Liu

**Affiliations:** ^1^ smARTLab, Department of Computer Science, University of Liverpool, Liverpool, United Kingdom; ^2^ Department of Engineering Mathematics and Bristol Robotics Laboratory, University of Bristol, Bristol, United Kingdom; ^3^ inte-R-action Lab and Centre for Autonomous Robotics (CENTAUR), University of Bath, Bath, United Kingdom; ^4^ Department of Mechanical Engineering and Materials Science, Yale University, New Haven, CT, United States; ^5^ Department of Computer Science and Technology, Tsinghua University, Beijing, China

**Keywords:** tactile sensing, robot perception, editorial, robot sensing and perception, robot learning and control

## 1 Introduction

Animals interact with the world through multimodal sensing inputs, especially vision and touch sensing in the case of humans interacting with our physical surroundings. In contrast, artificial systems usually rely on a single sensing modality, with distinct hardware and algorithmic approaches developed for each modality. For example, computer vision and tactile robotics are usually treated as distinct disciplines, with specialist knowledge required to make progress in each research field. Future robots, as embodied agents interacting with complex environments, should make best use of all available sensing modalities to perform their tasks.

Over the last few years, there have been advances in the fusing of information from distinct modalities and selecting between those modalities to use the most appropriate information for achieving a goal; for example grasping or manipulating objects in a desired manner, such as stacking objects in storage crates or folding clothing. We have seen a shift in the ways of linking vision and touch, from combining hand-crafted features (Luo et al., 2015; Luo et al., 2017) to learning a shared latent space with deep neural networks (Luo et al., 2018; Lee et al., 2019a). The integration of the visual and haptic cues have been shown to enhance perception in one modality, either enabling better tactile understanding of haptic adjectives (Gao et al., 2016) or learning visual representations (Pinto et al., 2016), and also result in better performance in achieving a task (Luo et al., 2018; Lee et al., 2019b).

Another trend is that there has been a recent acceleration in the development of optical tactile sensors using cameras, such as the GelSight (Yuan et al., 2017a; Johnson and Adelson, 2009) and TacTip (Ward-Cherrier et al., 2018; Chorley et al., 2009), which bridge the gap between vision and tactile sensing to create cross-modal perception. On the one hand, this crossover has enabled techniques developed for computer vision to be applied to tactile sensing; examples include the use of convolutional neural networks for estimating contact variables directly from the tactile images (Yuan et al., 2017b; Lepora et al., 2019), and also sim-to-real methods that were pioneered in robot vision but are now finding application in robot touch (Fernandes et al., 2021). On the other hand, there have been the development of methods that connect the look and feel of objects being interacted with (Calandra et al., 2017), progressing more recently to methods that can transform between or match visual and tactile data (Takahashi and Tan, 2019; Lee et al., 2019a; Li et al., 2019).

## 2 Contents of the Research Topic

The contents of the Research Topic include four papers on topics addressing diverse challenges of multimodal and cross-modal perception with vision and touch.

In “A Framework for Sensorimotor Cross-Perception and Cross-Behavior Knowledge Transfer for Object Categorization,” Tatiya et al. propose a framework for knowledge transfer across exploratory behaviors, e.g., press, grasp and hold, and sensory modalities, with audio, haptic, vibrotactile and visual feedback used. They use two models based on variational auto-encoders and encoder-decoder networks respectively to map shared multi-sensory object observations of a set of objects across different robots. Their results of categorising 100 objects indicate that sensorimotor knowledge about objects can be transferred both across behaviors and across sensory modalities, which can boost the robot’s capability in cross-modal and cross-behavior perception.

Haptic information can also be conveyed to a user during teleoperation tasks in the form of visual cues. This cross-modal sensation between visual and tactile sensing is usually termed pseudo-haptics (Li et al., 2016). In “Proposal and Evaluation of Visual Haptics for Manipulation of Remote Machine System” by Haruna et al., this approach is assessed using electroencephalogram (EEG) data on a Virtual Reality (VR) setting. The authors carried out a human-subject experiment where users were asked to perform a series of pick-and-place tasks with and without displaying a visual overlay with haptic information. The main finding of this paper is that the added pseudo-haptic information improves the usability of teleoperation systems and reduces users’ cognitive load. Additionally, the results also suggest that the brainwave information flow can be used as a quantitative measurement of a system’s usability or “familiarity.”

In “Using Tactile Sensing to Improve the Sample Efficiency and Performance of Deep Deterministic Policy Gradients (DDPG) for Simulated In-Hand Manipulation Tasks”, Melnik et al. and co-authors demonstrate the importance of tactile sensing for manipulation with an anthropomorphic robot hand. They perform in-hand manipulations tasks such as reorienting a held block, using a simulation model of the Shadow Dexterous Robot Hand covered with 92 virtual touch sensors. Deep reinforcement learning methods such as DDPG are known to require a large number of training samples that can make them impractical to train in physical environments. The authors showed that tactile sensing data can improve sample efficiency up to three-fold with a performance gain of up to 46%, on several simulated manipulation tasks.

GelTip is an optical tactile sensor proposed by Gomes et al. in the work entitled “Blocks World of Touch: Exploiting the Advantages of All-around Finger Sensing in Robot Grasping.” This sensor is composed of a rounded fingertip fully covered with an elastomer and with a camera mounted at the sensor base. The deformations of the elastomer that occur when the fingertip touches an object are captured and precisely tracked by the camera. The geometry of the sensor and location of the camera allows the device to detect deformations from any position on the fingertip, which contrasts with the reduced contact region found in traditional optical sensors. GelTip can identify contact location processes with 1 mm precision. This sensor has been mounted on a robotic gripper and successfully tested with object touch and grasping tasks. This process has shown that the sensor is capable of detecting collisions from any orientation while approaching to an object, which can be used by the robotic gripper to the grasping strategy. Overall, GelTip offers an effective optical tactile sensor with the potential to enable many robotic gripping and manipulation tasks that require tactile sensing from all over the fingertip.

## References

[B1] Calandra R. Owens A. Upadhyaya M. Yuan W. Lin J. Adelson E. H. (2017). “The Feeling of Success: Does Touch Sensing Help Predict Grasp Outcomes?,” in Conference on Robot Learning, Mountain View, CA, November 13–15, 2017, 314–323.

[B2] Chorley C. Melhuish C. Pipe T. Rossiter J. (2009). “Development of a Tactile Sensor Based on Biologically Inspired Edge Encoding,” in IEEE International Conference on Advanced Robotics, Munich, Germany, June 22–26, 2009 (IEEE), 1–6.

[B3] Fernandes D. G. Paoletti P. Luo S. (2021). Generation of GelSight Tactile Images for Sim2Real Learning. IEEE Robot. Automat. Lett. 6, 4177–4184. 10.1109/LRA.2021.3063925

[B4] Gao Y. Hendricks L. A. Kuchenbecker K. J. Darrell T. (2016). “Deep Learning for Tactile Understanding from Visual and Haptic Data,” in IEEE International Conference on Robotics and Automation, Stockholm, Sweden, May 16–May 21, 2016 (IEEE), 536–543.

[B5] Johnson M. K. Adelson E. H. (2009). Retrographic Sensing for the Measurement of Surface Texture and Shape. In IEEE Conference on Computer Vision and Pattern Recognition, Miami, FL, United States, June 20–June 25, 2009 (IEEE), 1070–1077.

[B6] Lee J.-T. Bollegala D. Luo S. (2019a). ““Touching to See” and “Seeing to Feel”: Robotic Cross-Modal Sensory Data Generation for Visual-Tactile Perception,” in IEEE International Conference on Robotics and Automation (IEEE), Montreal, Canada, May 20–24, 2019 (IEEE), 4276–4282.

[B7] Lee M. A. Zhu Y. Srinivasan K. Shah P. Savarese S. Fei-Fei L. (2019b). “Making Sense of Vision and Touch: Self-Supervised Learning of Multimodal Representations for Contact-Rich Tasks,” in IEEE International Conference on Robotics and Automation (IEEE), Montreal, Canada, May 20–24, 2019 (IEEE), 8943–8950.

[B8] Lepora N. F. Church A. De Kerckhove C. Hadsell R. Lloyd J. (2019). From Pixels to Percepts: Highly Robust Edge Perception and Contour Following Using Deep Learning and an Optical Biomimetic Tactile Sensor. IEEE Robot. Autom. Lett. 4, 2101–2107. 10.1109/lra.2019.2899192

[B9] Li M. Sareh S. Xu G. Ridzuan M. B. Luo S. Xie J. (2016). Evaluation of Pseudo-haptic Interactions with Soft Objects in Virtual Environments. PLoS One 11, e0157681. 10.1371/journal.pone.0157681 27352234 PMC4924842

[B10] Li Y. Zhu J.-Y. Tedrake R. Torralba A. (2019). “Connecting Touch and Vision via Cross-Modal Prediction,” in IEEE/CVF Conference on Computer Vision and Pattern Recognition (IEEE), Long Beach, CA, June 16–20, 2019 (IEEE). 10609–10618.

[B11] Luo S. Bimbo J. Dahiya R. Liu H. (2017). Robotic Tactile Perception of Object Properties: A Review. Mechatronics 48, 54–67. 10.1016/j.mechatronics.2017.11.002

[B12] Luo S. Mou W. Althoefer K. Liu H. (2015). “Localizing the Object Contact through Matching Tactile Features with Visual Map,” in IEEE International Conference on Robotics and Automation, Seattle, WA, United States, May 26–May 30, 2015 (IEEE), 3903–3908.

[B13] Luo S. Yuan W. Adelson E. Cohn A. G. Fuentes R. (2018). ViTac: Feature Sharing between Vision and Tactile Sensing for Cloth Texture Recognition, IEEE International Conference on Robotics and Automation, Brisbane, Australia, May 21–25, 2018 (IEEE), 2722–2727.

[B14] Pinto L. Gandhi D. Han Y. Park Y.-L. Gupta A. (2016). “The Curious Robot: Learning Visual Representations via Physical Interactions,” in European Conference on Computer Vision, Amsterdam, Netherlands, October 10–16, 2016. Editors Leibe B. Sebe N. Welling M. Matas J. (Amsterdam: Springer Verlag), 3–18.

[B15] Takahashi K. Tan J. (2019). “Deep Visuo-Tactile Learning: Estimation of Tactile Properties from Images,” in IEEE International Conference on Robotics and Automation, Montreal, QC, Canada, May 20–May 24, 2019 (IEEE), 8951–8957.

[B16] Ward-Cherrier B. Pestell N. Cramphorn L. Winstone B. Giannaccini M. E. Rossiter J. (2018). The TacTip Family: Soft Optical Tactile Sensors with 3D-Printed Biomimetic Morphologies. Soft Robotics 5, 216–227. 10.1089/soro.2017.0052 29297773 PMC5905869

[B17] Yuan W. Dong S. Adelson E. H. (2017a). Gelsight: High-Resolution Robot Tactile Sensors for Estimating Geometry and Force. Sensors 17, 2762. 10.3390/s17122762 PMC5751610 29186053

[B18] Yuan W. Zhu C. Owens A. Srinivasan M. A. Adelson E. H. (2017b). “Shape-independent Hardness Estimation Using Deep Learning and a Gelsight Tactile Sensor,” in IEEE International Conference on Robotics and Automation (IEEE), Singapore, May 29–June 3, 2017 (IEEE), 951–958.

